# Behavioural elements and sensory cues involved in sexual isolation between *Drosophila melanogaster* strains

**DOI:** 10.1098/rsos.172060

**Published:** 2018-05-09

**Authors:** Micheline Grillet, Jean-François Ferveur, Claude Everaerts

**Affiliations:** Centre des Sciences du Goût et de l'Alimentation, AgroSup Dijon, CNRS, INRA, University Bourgogne Franche-Comté, 21000 Dijon, France

**Keywords:** incipient speciation, wing, leg, antenna, ovipositor extrusion, social interaction

## Abstract

Sensory cues exchanged during courtship are crucial for mate choice: if they show intraspecific divergence, this may cause or reinforce sexual isolation between strains, ultimately leading to speciation. There is a strong asymmetric sexual isolation between *Drosophila melanogaster* females from Zimbabwe (Z) and males from all other populations (M). While M and Z flies of both sexes show different cuticular pheromones, this variation is only partly responsible for the intraspecific isolation effect. Male acoustic signals are also partly involved in sexual isolation. We examined strain-specific courtship behaviour sequences to determine which body parts and sensory appendages may be involved in sexual isolation. Using two strains representative of the Z- and M-types, we manipulated sensory cues and the social context; we then measured the consequence of these manipulations on courtship and copulation. Our data suggest that Z females mated best with males whose sensory characteristics matched those of Z males in both quantity and quality. M females were less choosy and much less influenced by the sensory and social contexts. Differences in emission and reception of sensory signals seen between Z and M flies may lead to the concerted evolution of multiple sensory channel, thereby shaping a population-specific mate recognition system.

## 1. Introduction

Sensory signals exchanged between individuals of most vertebrate and invertebrate species are crucial for guiding intraspecific interactions, thereby allowing mate recognition and social organization [1–4]. Insects, which often depend on such signals and generally have a short generation time, are suitable model organisms to study the evolution of sensory communication. For example, the alteration of a single gene coding for an enzyme involved in the production of moth pheromones has led to the appearance of a novel species [5,6]. This implies that, during speciation, genetic factors coding for the emission and reception of sensory signals should remain tightly linked, in order to avoid the collapse of sensory communication. Where the emission–reception link of a single sensory signal does initially diverge, this may be followed by the secondary changes that reinforce the incipient isolation between diverging populations or sub-species [7]. This would explain why examples of speciation rarely turn out to be based on the variation of a single signal but rather depend on several signals acting in synergy (in human [8], mouse [9] and cockroach [10]). This suggests that each species possesses a specific mate recognition system (SMRS) combining different sensory modalities, each of which has a species-specific weight in the recognition/isolation process [11,12].

*Drosophila* constitutes an ideal group of species for studying the role of multiple sensory signals on mate recognition and sexual isolation: their social and sexual interactions depend on vision, sound, olfaction and taste [13–22]. The courtship of *Drosophila melanogaster* flies consists of a series of stereotyped behaviours [23,24]. While male courtship behaviour largely follows a genetically fixed action pattern [25], it can be modulated by several female behaviours that are correlated with copulation success [26–28]. A useful way of investigating the impact of sensory modalities in species-specific recognition involves comparing the mating patterns of geographically distant populations of the same species [29]. For example, a strong incipient speciation process has been described between *D. melanogaster* populations found in Zimbabwe (Z strains) and non-overlapping populations from all other geographical regions (M strains). In particular, a strong asymmetric isolation has been observed, whereby a very low copulation frequency is observed between Z females and M males (less than 10% matings in 60 min compared with 60–80% in homotypic pairs) [30,31]. This incipient sexual isolation has been attributed to a small number of genetic loci that have been roughly mapped on the autosomes of females and males (three and four loci, respectively). These loci may have concurrently evolved during intraspecific divergence [32]. The *desat2* gene, coding for a Δ9-desaturase thought to be expressed only in Z strains, may be one of the factors involved in this example of incipient speciation [33,34]. A second Δ9-desaturase gene*, desat1*, which is tightly linked to *desat2*, controls both the emission and the perception of cuticular pheromones [35,36]. This suggests that the strong sexual isolation between M and Z flies may be due to the interaction between *desat1*, *desat2* and other mating-related genes [30,37,38]. Some of the sensory cues, diverging between Z and M strains and potentially involved in the strong isolation effect, have been evaluated [30,39]. Cuticular pheromones and/or acoustic signals are partly—but not totally—responsible for the sexual isolation between M males and Z females [30,40].

We hypothesized that multiple sensory cues, their combination and their quantitative variation, could explain isolation. To precisely characterize the sensory signals involved in sexual isolation between Z females and M males, we carried out a fine-grain characterization of courtship behaviour in copulating and non-copulating pairs. We then manipulated some of the body parts potentially linked to courtship variation and involved in the production and/or reception of sensory cues. In some of these experiments, we also changed the social environment of the flies.

## 2. Material and methods

### 2.1. Strains

The Zimbabwe Z30 line (hereafter Z6 (the Z strain used here), as in [30]) and the M-type Arkansas-Louisiana line (AL) [30,41] were provided by Prof. Jerry Coyne (University of Chicago). These inbred strains were chosen based on their differences in copulation pattern and cuticular pheromones [30]. Flies were raised in 150 ml glass vials containing 30 ml of yeast/cornmeal/agar medium and were kept at 24.5 ± 0.5°C with 65 ± 5% humidity on a 12 L : 12 D cycle (subjective day 8.00–20.00). Adults were transferred every two/three days to avoid larval competition and to provide sufficient progeny for testing. Flies were sexed under brief CO_2_ anaesthesia 0–4 h after emergence. Males were individually kept in fresh glass food vials for 4 or 5 days before testing; females were kept in single-sex groups of five to ten flies of the same age. Each test was performed over several days and took place 1–4 h after lights on. All experiments were performed under similar conditions.

### 2.2. Behaviour

We did not use genetically manipulated flies for two reasons: (1) mutations and transgenes often induce undesired pleiotropic effects [17,42], and (2) it is difficult to introduce a sensory mutation into the background of a Z wild-type strain while maintaining the linkage of the courtship-associated alleles that determine the highly polygenic Z behavioural phenotype [32]. All courtship and copulation tests were performed under a 1.6 cm^3^ watch glass used as a courtship observation chamber, except for the video and social context experiments. Behavioural observations were always performed from 1 to 4 h after lights on.

#### 2.2.1. Locomotor activity of single flies

To determine whether differences in sexual behaviour could be due to differences in general activity, we compared locomotor activity in single female and male flies of the two strains under white and red light (25 W, Kodak ‘Safe-light’ #1 filter; red light wavelength 590–750 nm versus 390–750 nm for white light [43]). Single naive flies were introduced into the observation chamber that was placed over a grid. After a 10 min adaptation period, the fly's locomotor activity was quantified by counting the number of lines crossed during a total of 50 s (5 × 10 s periods with a 50 s interval between each period).

#### 2.2.2. Courtship and copulatory behaviours

Tests performed under white or red light lasted either 10 or 60 min (see below). Depending on the experiment, all or some of the following female × male fly pairs were tested: AL × AL, Z6 × AL, AL × Z6 and Z6 × Z6. A single male was aspirated without anaesthesia into the observation chamber. After 10 min, a virgin female (intact or decapitated) was introduced and we observed male activity and noted both courtship latency and, in the case of intact females, copulation latency. Latency was defined as the interval between introduction into the chamber and behaviour onset. Observations lasted 10 min or until copulation occurred. We calculated the courtship index (percentage of time spent by the male courting the female during the observation period). Pairs were kept together for 60 min to estimate courtship and copulation frequencies; if courtship or copulation did not occur, the observation was used only for the calculation of frequencies, not latencies. The test involving intact females allowed us to estimate female receptivity (acceptance of the male); decapitated females cannot produce active acceptance or rejection signals and never copulate. Virgin CO_2_-anaesthetized 4-day-old females were decapitated with a Gillette Double Edge Platinum razor blade, 1 h before the test. Decapitated females remained alive for 3 to 4 h but were mostly immobile.

#### 2.2.3. Video analysis of locomotion and courtship behaviours

The behaviour of Z6 × Z6 and Z6 × AL pairs was videotaped for 10 min or until copulation occurred, between 2 and 4 h after lights on. A Panasonic AJ-D230E digital video tape recorder and a Sony SSC-DC38P colour digital video camera were used, connected to a Leica MZ8 binocular microscope. Behaviour was recorded for either 10 min or until copulation occurred. Movies were analysed with Observer Video-Pro 4.0 software (Noldus Information Technology, The Netherlands [44]) on a PC fitted with a PC–VITC Time Code Reader (Adrienne Electronics Corporation, USA) and with a Screen Machine II video overlay board (FAST Multimedia AG, Germany) with a 0.04 sec accuracy, following the description in [24]. Briefly, each state and elementary behaviour (each complex behaviour consisting of a set of elementary behaviours) was noted, and the proportion of time performing that behaviour, relative to the total observation time (expressed as the mean ± s.e.m. [28]), was noted. We also analysed videotaped behaviours to measure locomotor activity: a transparent sheet divided into six equal areas was placed on the video monitor screen, and we scored the number of boundaries crossed by each fly of the tested pairs. Activity during the observation period was measured as the number of boundaries crossed during each one-eighth fraction of the total observation period for each pair; the duration of each fraction of the period was relative, not absolute and could have a different length for different pairs [28].

### 2.3. Manipulating sensory modalities

#### 2.3.1. Vision

*Drosophila melanogaster* is a light-independent species [45] which mates less frequently under darkness than under light [46]. To determine strain-specific light-dependence in both Z6 and AL flies, we compared their sexual behaviour under white and red lights.

#### 2.3.2. Chemosensation/audition

In one experiment, we performed bilateral ablation of the maxillary palps (in females), aristae (in males) or funiculi (third antennal segment carrying the arista; in both sexes). All surgical ablations were carried out with Moria Worst Microscissors (#17000) on CO_2_-anaesthetized flies, under a Leica Wild MZ8 stereomicroscope. Operated flies were kept in food vials for 24 h until the behavioural test. Non-operated control flies were also CO_2_-anaesthetized and kept in similar conditions.

In another experiment, we manipulated the wings, which are involved in the emission of acoustic signals [47] and in the diffusion and perception of chemosensory signals [16,48,49]. Based on previous studies that showed the influence of different parts of the male wing on copulation [50,51], we tested the effect of different types of wing ablation in males: (1) complete bilateral ablation (cutting next to the wing articulation on the thorax; ‘*Bilateral’*); (2) complete unilateral ablation (‘*Unilateral*’); (3) bilateral ablation of the distal region (cutting beyond the posterior crossvein; ‘*Distal’*); (4) bilateral ablation of the posterior margin (from longitudinal vein L4 to anal vein A2; ‘*Posterior margin’*); (5) bilateral ablation of the costal margin (cutting from longitudinal vein L4 to radial vein R1; ‘*Anterior margin’*).

### 2.4. Manipulation of the social context and contact duration

To test the effect of social context on sexual isolation between Z6 females and AL males, we either changed the number of male flies placed with a single female, and/or we altered the male capacity to produce sensory cues using a regular copulation chamber. To test the effect of additional olfactory and auditory cues provided by extra flies without contact with the focal pair, we used a double-floor chamber separated by a mesh (electronic supplementary material, figure S1).

#### 2.4.1. Competition between two males

The performance of two intact AL and Z6 males placed with one female (AL or Z6) was measured under both red and white light. Both males were introduced into the observation chamber 10 min after the female. To distinguish the males, we slightly clipped (0.5 mm) one wing (alternately for each male genotype), a procedure which does not affect *Drosophila* copulation success [52,53]. In each test, the genotype of the copulating male was noted together with its copulation latency.

We also placed an intact male (AL or Z6) with a second male of the other genotype that was unable to copulate. Male copulation impairment was achieved by depositing a tiny droplet of Loctite^®^ Super-Glue on the male genitalia using a needle. Males were held still with a small brush until the glue was dry. Single Z6 females were also placed with an intact AL male and a manipulated-wing Z6 male (see above). All surgery was performed under a binocular microscope on CO_2_-anaesthetized flies, 24 h before testing.

#### 2.4.2. Increasing male number (1–10)

The effect of the presence of 1 to 10 virgin male(s) of the same strain on the copulation success with single homo- or heterotypic virgin females was measured under red light. In this case, males were introduced in the observation chamber 10 min after the female.

#### 2.4.3. Effect of the social context without physical contact

To test the role of olfactory and auditory stimuli produced by flies physically separated from the focal pair, we used a copulation chamber made of two parts separated by a fine nylon mesh (electronic supplementary material, figure S1; see above). In the upper part, a Z6 female was paired with an intact male (Z6 or AL), while five Z6 males, or five AL males, or 2 AL × AL pairs were placed in the lower part of the chamber. In all these experiments, the overall copulation frequency and latency of each focal pair or genotype were recorded over a 60 min period.

#### 2.4.4. Longer periods of contact between intact pairs

To evaluate whether sexual isolation can be affected by longer periods of contact, homo- and heterotypic pairs of male and female flies were kept together in fresh food vials for either 24 or 48 h. Males were then discarded and the number of vials containing at least one adult progeny was noted 2 weeks later. This allowed us to estimate the frequency of fertile pairs.

### 2.5. Statistics

Courtship and copulation frequencies were recorded during the first 10 min or 60 min and compared using Wilks *G*^2^ likelihood ratio tests completed with a computation of significance by cell (Fisher's test). Proportions were compared using a Marascuilo procedure at *p* = 0.05. Courting and copulation latencies, courtship and locomotor indices and video data were compared using Kruskall–Wallis tests completed by a Conover–Iman multiple pairwise comparison (at level *p* = 0.05), after excluding extreme outliers using Tukey's method ([54]; maximum = 6% of the sample). Statistical analyses were conducted with XLSTAT 2012 [55]. For each test, the sample size is indicated in the figure legend.

## 3. Results

### 3.1. Precise characterization of courtship behaviour

**Figure 1.**
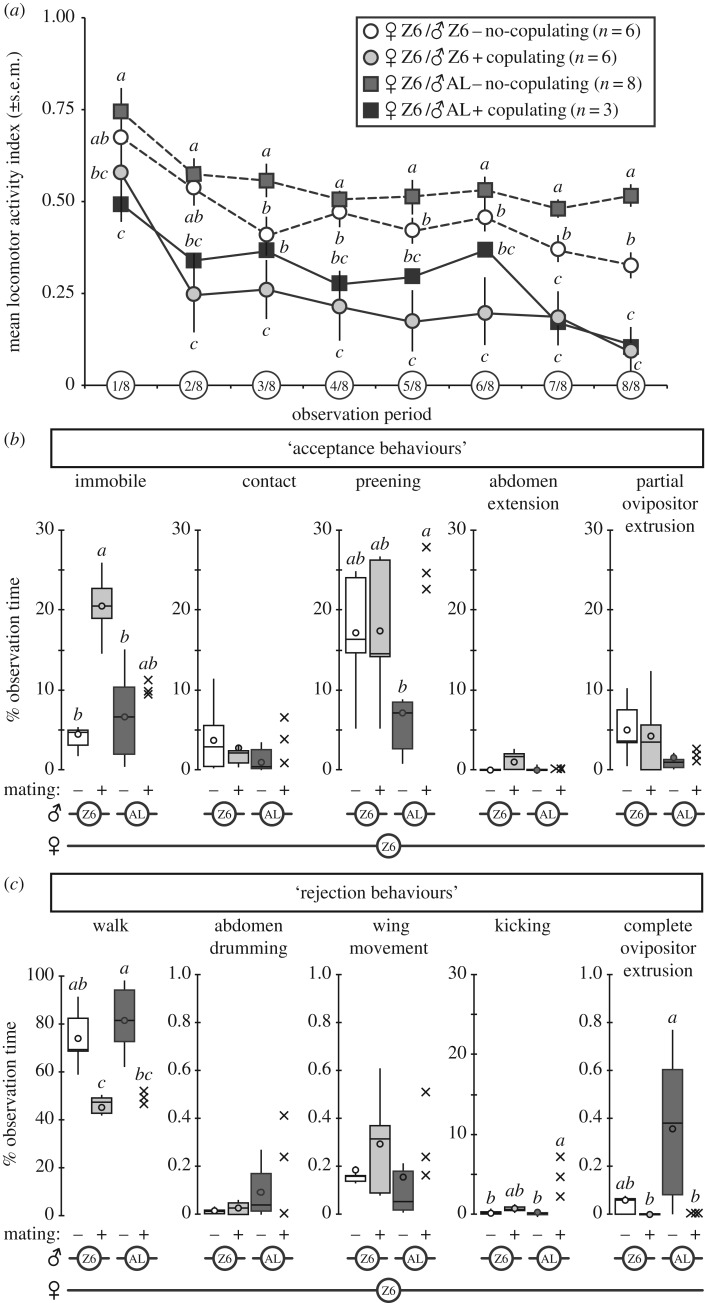
(*Overleaf*.) Precise characterization of courtship behaviour. Mean locomotor activity (*a*) was measured in mating and non-mating homo- and heterotypic pairs consisting of one Z6 female and one Z6 or AL male. This measure was calculated for both flies, for each one-eighth of the total observation period (see Material and methods). For each period, mean locomotor activity was compared using a Kruskal–Wallis test, completed by Conover–Iman's multiple pairwise comparison (two-tailed with Bonferroni correction). Sample sizes are indicated on the figure; letters indicate statistically significant differences. Female behavioural components that predicted acceptance (*b*) or rejection (*c*) of the male were estimated as proportions of the observation time in homo- and heterotypic pairs consisting of one Z6 female and one Z6 or AL male, depending on mating (+, −; indicated at the bottom of the graphs). These proportions of active time are shown as box-and-whisker plots indicating the 25th and 75th percentiles (Q1 and Q3; boxes), the median (line within box) and the limits (whiskers) beyond which values were considered anomalous (these limits were calculated as follows: lower limit = Q1 − 1.5 × [Q3 − Q1] and upper limit = Q1 = 1.5 × [Q3 − Q1]) and were compared using a Kruskal–Wallis test. The letters above the whiskers indicate statistically significant differences.

To decipher the behavioural events potentially involved in sexual isolation between Z-type females and M-type males, we precisely characterized the locomotor activity and the sexual interaction of Z6 females paired either with AL males (Z6 × AL pairs), or with homotypic males (Z6 × Z6 pairs; figure 1).

#### 3.1.1. Locomotor activity

In each pair, the locomotor activity of the two flies over the time course of their courtship was highly positively correlated (*r^2^* range: 0.69–0.978; *p* range: < 10^−4^−0.049; see electronic supplementary material, figure S3A). The locomotor activity of each pair was measured during eight periods of equal duration (1/8 to 8/8; the duration of these periods was calculated relative to copulation onset and therefore varied between pairs; figure 1*a*). A significant difference between copulating and non-copulating pairs was observed very rapidly after courtship initiation (1/8 and 2/8 periods in Z6 × AL and Z6 × Z6 pairs, respectively; Kruskal–Wallis test: range with *K_3df_* = 14.4–19.6, *p* =  < 10^−4^–0.002). Immediately after these periods, the locomotor activity of pairs that eventually copulated decreased and remained lower than that observed in pairs that did not copulate. This difference was maintained until copulation. In single flies observed alone, locomotor activity was generally lower in Z6 flies compared with AL flies of the same sex (electronic supplementary material, figure S2). This difference was observed under both white (W) and red light (R; *K_3df_* *=* 23.6, *p* < 10^−4^ and *K_3df_* *=* 39.3, *p* < 10^−4^ for females and males, respectively; *n* = 29–30).

#### 3.1.2. Behavioural elements

To detect the behavioural elements potentially involved in the copulation success of Z6 × AL and Z6 × Z6 pairs, we used video recording to measure the total duration of several precise behavioural elements shown by Z6 females. Some of these behaviours, which are assumed to predict the acceptance or rejection of the male partner, significantly differed according to whether the pair eventually copulated or not (figure 1*b*,*c*). In Z6 × AL pairs, Z6 females that copulated spent more time preening (*K_3df_* = 8.2, *p* = 0.042) and kicking (*K_3df_* = 10.8, *p* = 0.013), but less time walking (*K_3df_* = 14.3, *p* = 0.002) and completely extruding their ovipositor (*K_3df_* = 9.8, *p* = 0.020), compared to Z6 females that did not copulate. A closer examination of the body parts involved in preening revealed several differences according to whether the female eventually copulated or not (electronic supplementary material, figure S3B). In both kinds of pairing, females that eventually mated showed a higher relative duration of head preening; the only effect seen for wing preening was a decrease observed in Z6 × AL pairs that did not copulate. The two kinds of pair showed different effects for leg preening: Z6 females that eventually copulated showed increased leg 1 (prothoracic) preening and decreased leg 2 (mesothoracic) preening when paired with AL males, whereas they decreased leg 1 and increased leg 3 preening when paired with Z6 males.

In Z6 × Z6 pairs, Z6 females that eventually mated spent a longer period immobile (not moving and not showing any other visible behaviour; *K_3df_* = 12.0, *p* = 0.007) and they walked less than females that did not copulate (*K_3df_* = 14.3, *p* = 0.002). In summary, the comparison of the two pairs suggests that copulation in Z6 × AL pairs occurred more frequently when Z6 females showed more preening and kicking but less complete ovipositor extrusion.

### 3.2. Role of visual signals, locomotion and female receptivity

**Figure 2.**
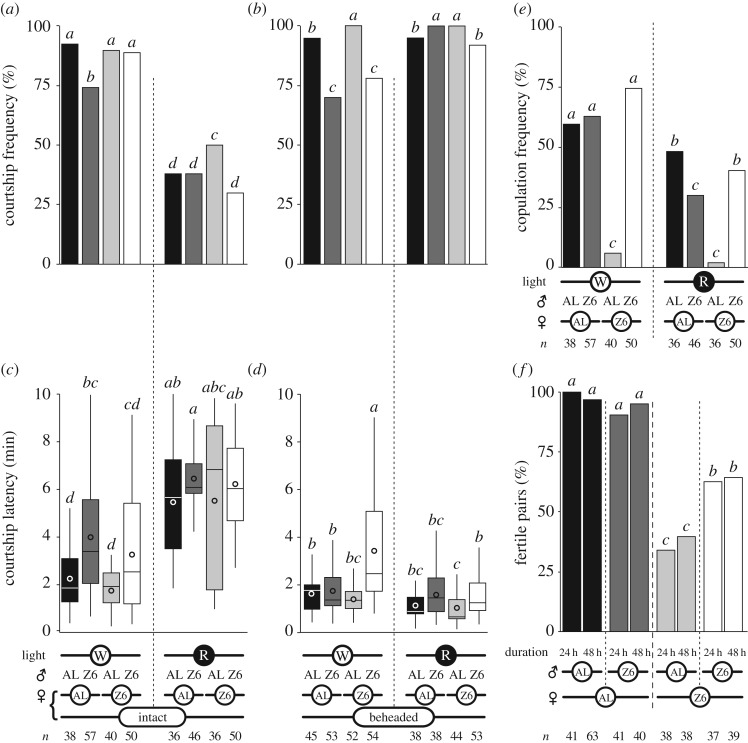
Effect of light and contact duration. Bars indicate the frequency of courtship during a 10 min period (*a*,*b*) and copulation (*e*) measured in homo- and heterotypic pairs consisting of Z6 and/or AL flies, under white (W) or red light (R) (indicated at the bottom of the graphs) over a 60 min period. Sample sizes are indicated at the bottom of the graphs. Courtship of intact or decapitated females is shown at the bottom of the graphs. Frequencies were compared using a Wilks *G*^2^ likelihood ratio test completed with a computation of significance by cell (Fisher's test; letters above bars indicate statistically significant differences). Courtship latency in these pairs is shown (*c*,*d*), represented with box-and-whisker plots to indicate the 25th and 75th percentiles (Q1 and Q3; boxes), the median (line within box) and the limits (whiskers) beyond which values were considered anomalous and excluded according to Tukey's method (these limits were calculated as follows: lower limit = Q1 − 1.5 × [Q3 − Q1] and upper limit = Q1 = 1.5 × [Q3 − Q1]). Courtship latencies were compared with Kruskal–Wallis tests. Letters above the whiskers indicate statistically significant differences. The frequency of females with progeny in homo- and heterotypic pairs kept in food vials for 24 or 48 h is shown in (*f*).

To investigate the sensory basis of the strong relationship between a decline in locomotor behaviour and successful copulation (figure 1*a*), we suppressed their visual perception of motion-related signals. By definition, female locomotion was suppressed in decapitated females, although mating could not occur in these flies. The behaviour of all four female × male pairs (AL × AL, AL × Z6, Z6 × Z6 and Z6 × AL) was observed under white light and red light. Male courtship indices were also compared between intact (figure 2*a*,*c*,*e*) and decapitated females (figure 2*b*,*d*).

Under white light (W), Z6 males showed a reduced courtship frequency with all females except their own intact females (figure 2*a*; Wilks *G*^2^ likelihood ratio test: $G_{7df}^{2}=110.4$, *p* < 10^−4^). In particular, they showed a similarly decreased courtship frequency with both types of decapitated female (figure 2*b*; $G_{7df}^{2}=50.1$, *p* < 10^−4^). Compared with other pairs, Z6 males showed less frequent courtship with both intact and decapitated AL females (figure 2*a*,*b*; Wilks *G*^2^ likelihood ratio test: $G_{7df}^{2}=110.4$, *p* < 10^−4^). By contrast, AL males courted intact Z6 females slightly more often under red light (R) and decapitated Z6 females under W, compared with other pairs ($G_{7df}^{2}=50.1$, *p* < 10^−4^). The courtship frequency with intact females generally decreased under red light; no such effect occurred with decapitated females. The courtship index with decapitated females showed no or only a slight reduction under red light, but strongly decreased in the case of intact females (*K_7df_* = 124.4, *p* < 10^−4^; electronic supplementary material, figure S4).

During the 60 min observation period, very few Z6 × AL pairs copulated under either R or W conditions (figure 2*e*). Copulation latency was delayed in Z6 × Z6 pairs compared with AL × AL pairs, independent of light condition (electronic supplementary material, figure S4C). Under R, copulation was delayed in all pairs and AL × Z6 pairs mated less frequently than under W (figure 2*e* and electronic supplementary material, figures S4C; $G_{7df}^{2}=239.4$, *p* < 10^−4^ and *K_6df_* = 48.3, *p* < 10^−4^, respectively; *n* = 32–125). We also measured the fertility (ability to leave at least one progeny) of pairs kept in contact for either 24 or 48 h (figure 2*f*). The fertility frequency of AL × AL and AL × Z6 pairs significantly increased compared to the copulation frequency observed during 1 h ($G_{7df}^{2}=118.8$, *p* < 10^−4^, *n* = 37–63). This indicates that the great majority of pairs that did not copulate during the first hour (figure 2*e*) mated during the next 23 h. Similarly, the fertility of Z6 × AL pairs kept in contact for 24 and 48 h was much higher compared to their copulation frequency during the first hour of contact (we used the Marascuilo procedure, with significance set at *p* < 0.05, to compare the two independent datasets). The frequency of fertile Z6 × Z6 pairs was similar to their 60 min copulation frequency (figure 2*e*,*f*). To summarize, while impairment of visual cues did not increase copulation frequency in Z6 × AL pairs as measured over 60 min, mating frequency did significantly increase over the subsequent 23 h period.

### 3.3. Manipulating sensory cue production and perception

To evaluate the role of sensory cues exchanged during courtship, including those potentially involved in the fine behavioural differences noted above (figure 1), we manipulated some of the tissues and appendages potentially involved in the production and/or perception of these cues.

#### 3.3.1. Female wings and legs

**Figure 3.**
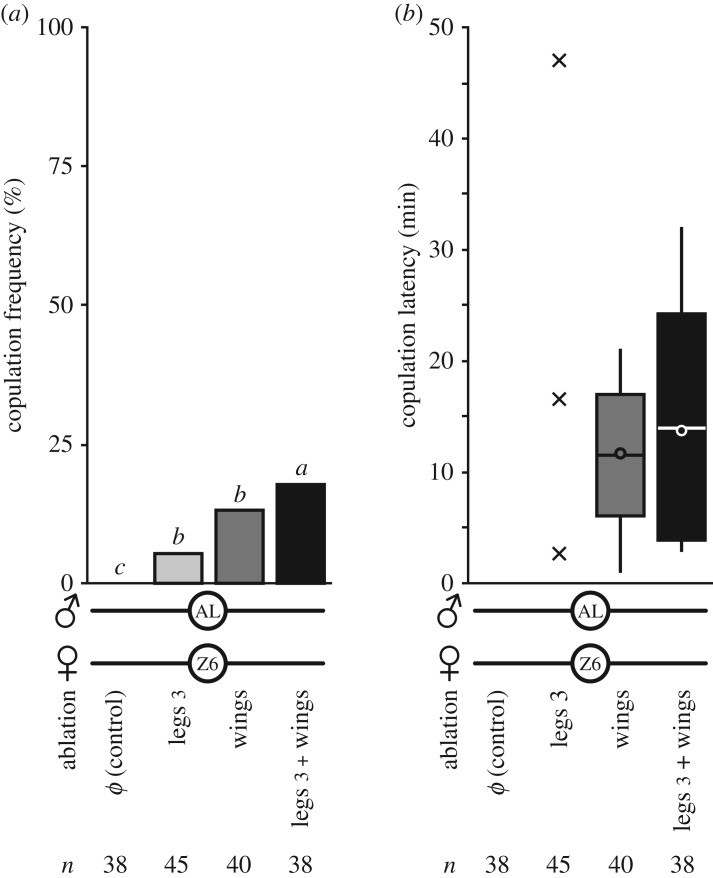
Ablation of wings and/or metathoracic legs. Copulation frequency (*a*) and copulation latency (*b*) of an intact AL male paired with a Z6 female without wings and/or metathoracic legs (legs 3). Controls were CO_2_ anaesthetized. For further details, figure 2.

Given the role of female wings and metathoracic legs (legs 3) in copulation success [28], we ablated these appendages in Z6 females to evaluate their role in the copulation of Z6 × AL pairs (figure 3). While copulation frequency significantly increased in Z6 females deprived of either wings or legs 3, this frequency slightly increased in females deprived of both wings and metathoracic legs (figure 3*a*; $G_{3df}^{2}=11.2$, *p* = 0.011, *n* = 38–40). These manipulations did not affect copulation latency (figure 3*b*).

#### 3.3.2. Chemosensory and/or auditory appendages

**Figure 4.**
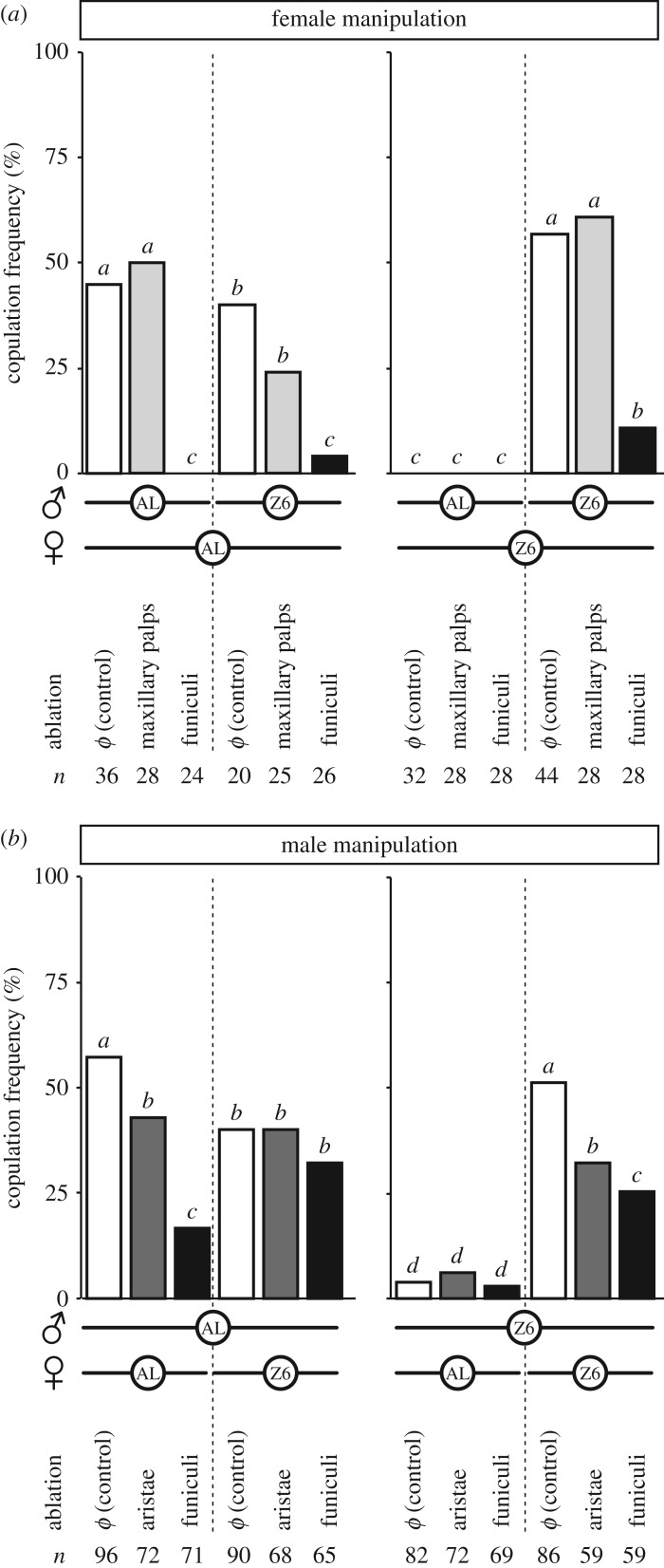
Ablation of head sensory appendages. Bars indicate copulation frequencies measured in pairs consisting of a manipulated female and an intact male (*a*) or a manipulated male and an intact female (*b*). Females’ maxillary palps or funiculi and male funiculi or aristae were ablated under CO_2_. Control flies were CO_2_ anaesthetized. For statistics, figure 2.

As the cuticular pheromones that vary between Z6 and AL flies are involved in sexual isolation, we ablated the chemosensory appendages potentially involved in their detection. In females, the maxillary palps or the funiculi—both involved in olfactory perception—were bilaterally ablated (figure 4*a*; note that funiculi, which carry the aristae, feather-like organs involved in auditory perception, have been studied in females [28]). In males, both funiculi, or only aristae, were ablated (figure 4*b*). In both sexes, ablation of the funiculi strongly decreased copulation frequency ($G_{5df}^{2}$ **ranging from 28.2 to 96.8, *p* < 10^−4^, *n* = 20–96). The two other types of ablation had much less or no effect on copulation frequency: females without maxillary palps were not affected, while males without aristae showed only a moderate decrease in mating with homotypic females (in AL × AL and Z6 × Z6 pairs). Note that the manipulation of Z6 males induced a significant effect on their copulation frequency with homotypic but not heterotypic females.

### 3.4. Manipulating sensory cues and social interaction

Given the importance of both the quality and quantity of sensory cues exchanged during courtship, we performed five experiments to evaluate the influence of the interaction between sensory cues and social context, with a special focus on Z6 × AL pairs.

#### 3.4.1. With two intact males

**Figure 5.**
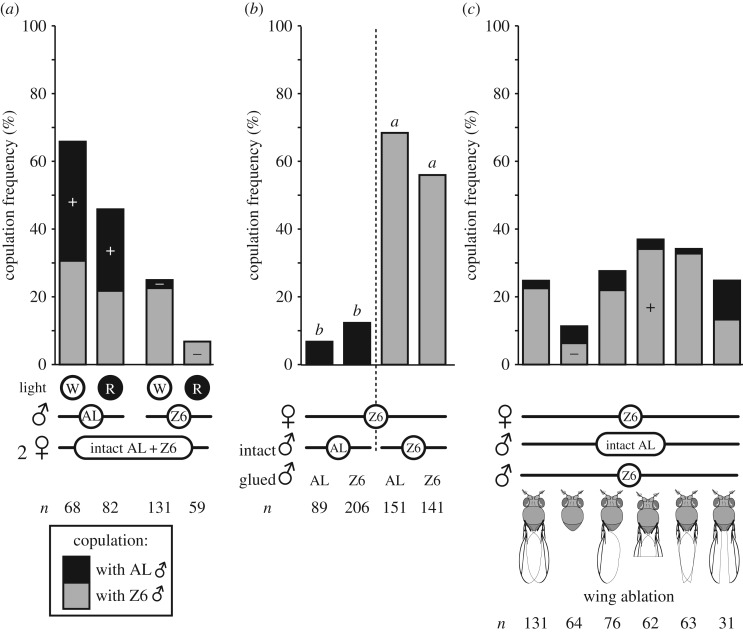
(*a*) Competition between two intact males. Stacked bar graphs represent the copulation frequencies of two intact AL and Z6 males (black and grey bars, respectively) competing for a single AL or Z6 female for 60 mins under white (W) or red light (R). The frequencies of non-copulating pairs and of mating Z6 and AL males were compared using a Wilks G^2^ likelihood ratio test completed with a computation of significance by cell (+ and − symbols indicate a significantly increased or decreased number of matings compared to the null hypothesis of no interaction between female type and light condition on mating success. (*b*) Interaction between one intact male and one genitalia-impaired male. We measured the performance of one intact male (AL or Z6) in the presence of one AL or Z6 male with glued genitalia and therefore unable to copulate. Copulation frequencies, represented by bars, were analysed using a chi-square test with a computation of significance by cell. Similar letters above bars indicate no significant difference. For statistics, Figure 5*a*. (*c*) Interaction between one intact AL male and one wing-manipulated Z6 male. Stacked bar graphs represent the copulation frequencies of an intact AL male competing with a Z6 wing-manipulated male for a single Z6 female. Wing ablations are explained beneath each graph. From left to right: control flies were CO_2_ anaesthetized; bilateral = total ablation of the both wings at the wing articulation; unilateral = total ablation of one wing—alternately right or left; distal part = ablation of the distal wing portion beyond the posterior crossvein; anterior margin = bilateral ablation of the costal wing margins from longitudinal vein L4 to radial vein R1; posterior margin = bilateral ablation of the wing posterior margins from longitudinal vein L4 to anal vein A2. For statistics, figure 2.

Significant copulation differences were noted when two intact males (Z6 and AL) were associated with each type of female either under W or R light (figure 5*a*; $G_{6df}^{2}=89.9$, *p* < 10^−4^, *n* = 59–131). When paired with AL females, both types of males showed a similar copulation success under both light conditions. Very few AL males copulated with Z6 females under W, while no AL male and few Z6 males copulated under R.

#### 3.4.2. With one intact male and one genitalia-altered male

To change male sensory stimuli without creating male–male competition for copulation, one intact male was associated with a second male (of either strain) that had glued genitalia and was therefore unable to copulate (figure 5*b*). All tests performed with Z6 females showed that the type of genitalia-impaired male did not affect the courtship success of the intact male ($G_{3df}^{2}=190.4$, *p* < 10^−4^, *n* = 89–206).

#### 3.4.3. With one intact AL male and one wing-manipulated Z6 male

Z6 females were associated with one intact AL male and one Z6 male with various degrees of wing ablation (figure 5*c*). The copulation success of Z6 males was significantly affected in two cases: they showed a drastic reduction after complete wing ablation and a slight increase with distal ablation ($G_{10df}^{2}=25.2$, *p* = 0.005, *n* = 31–131). No such effects were seen for AL males. The copulation frequency of AL males, which was always low, was similar to that of Z6 males deprived of their posterior wing margin.

#### 3.4.4. With an increasing number of males

**Figure 6.**
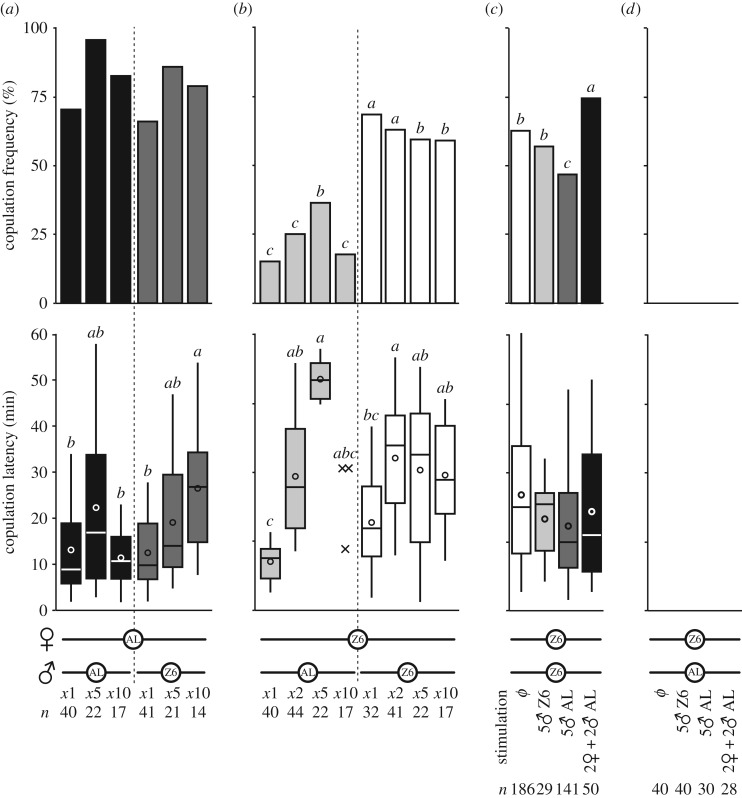
(*a*,*b*) Effect of male number. Mating frequencies (bar graph) and latencies (box-and-whisker plots) were measured in single AL (*a*) or Z6 females (*b*) paired for 1 hour with 1 to 10 AL or Z6 males. For statistics, figure 2. (*c*,*d*) Effect of fly stimuli without physical contact. Mating frequencies (bar graph) and latencies (box-and-whisker plots) were measured in focal intact pairs (Z6 × Z6 or Z6 × AL) placed in the upper floor of a two-part copulation chamber (see electronic supplementary material, figure S1) in the presence of various combinations of live intact flies in the lower part. For statistics, figure 2.

To increase the quantity of male sensory cues during male–male competition, a single female was associated with 1 to 10 males of the same strain (1, 5, 10 with AL female; 1, 2, 5, 10 with Z6 female). Female copulation was differentially affected depending both on (i) female and male strain and (ii) number of males. While AL female copulation frequency was not affected by male number, their copulation latency was delayed when placed with 10 Z6 males when compared with 10 AL males or with 1 Z6 male (figure 6*a*; *K_5df_* = 14.3, *p* = 0.014 254, *n* = 11–25). By contrast, Z6 females copulated more frequently but also more slowly with 5 AL males compared with lower or higher numbers of AL males (figure 6*b*; $G_{7df}^{2}=45.6$, *p* < 10^−4^, *n* = 17–46). Z6 females copulated less frequently with 5 or 10 Z6 males than with 1 or 2 Z6 males, but these matings were faster with 1 Z6 male than with 2 Z6 males (*K_7df_* = 35.8, *p* < 10–4, *n* = 6–27).

#### 3.4.5. Providing more fly stimuli without physical contact

Using divided mating chambers, we measured the behaviour of an intact focal pair of flies (either Z6 × Z6 or Z6 × AL) in the presence, or not, of various combinations of live intact flies placed in the lower part of the chamber (electronic supplementary material, figure S1). The copulation frequency of Z6 × Z6 pairs (i) decreased with five AL males, (ii) remained unchanged with five Z6 males and (iii) increased with two AL × AL pairs (figure 6*c*; $G_{5df}^{2}=14.9$, *p* = 0.003, *n* = 28–186). By contrast, no copulation occurred in Z6 × AL pairs whatever combination of flies was placed in the lower part of the observation chamber (figure 6*d*).

## 4. Discussion

Populations of the cosmopolitan *D. melanogaster* species were initially thought to share a similar SMRS [56,57]. Following the discovery of several exceptions to this panmictic rule [58–60], a marked case of sexual isolation was detected between Zimbabwe females (Z) and males from all other strains (M [31,61]). Previous explorations of the sensory cues involved in this strong asymmetric sexual isolation have revealed that strain-specific cuticular pheromones and, to a lesser extent, acoustic signals produced by male wings, are involved [30]. Here, we used complementary approaches to further unravel the behavioural and sensory bases of this example of incipient speciation.

We sought to precisely characterize the behavioural elements of the courtship of M males and Z females in order to detect subtle behavioural differences between copulating and non-copulating pairs. The comparison of these data with those shown by copulating and non-copulating Z × Z homotypic pairs allowed us to describe several behavioural elements that diverged in Z × M pairs, depending on whether they mated or not. These findings enabled us to map and manipulate some of the sensory tissue(s) or appendage(s) apparently involved. Another experiment involved manipulating choosy Z females either by changing (i) the number, genotype and/or sensory or behavioural phenotype of male partners (figures 4, 5 and 6*a*,*b*), or (ii) the quantity and quality of the sensory signals emitted in the presence of a single male (figure 6*c*,*d*).

### 4.1. Zimbabwe females’ choosiness: role of locomotion and visual cues

The asymmetric sexual isolation observed here clearly depends on the low sexual receptivity of Z6 females when presented with AL males, which nevertheless persisted in courting these females with a sustained intensity beyond the 1 h observation period (as indicated by their fertility frequency when kept in contact during 24 h; figure 2*f*; see below). The simultaneous decrease of locomotor activity shown by the two partners [28] was a good proxy for copulation success (figure 1*a*, electronic supplementary material, figure S3A). Indeed, when locomotor activity markedly decreased during the very early phases of courtship, this decrease lasted until copulation began. The locomotion of copulating Z6 × AL pairs diverged from that of non-copulating Z6 × Z6 pairs only during the last two phases of courtship (7/8 and 8/8).

The higher locomotor activity shown by single AL flies (electronic supplementary material, figure S2) may be partly involved in the sexual isolation: most AL males may have been unable to sufficiently reduce their locomotor activity to that of the Z6 female during the first two phases of courtship [28]. However, the fertility frequency observed in these pairs (and in all pairs involving AL females) kept together for 24 h indicates that an increased proportion of AL males copulated with Z6 females during the subsequent hours of contact (figure 2*f*). This could be the product of repeated copulation attempts by AL males [62,63]. This fits with the fact that single Z6 females copulated more frequently with five AL males than with a single AL male (figure 6*b*). The delayed copulation latency observed with five AL males may result of an increased male–male competition.

By contrast, more Z6 females copulated faster with a single Z6 male than with two Z6 males (figure 6*b*). The similarity between the 1 h copulation frequency and fertility (during 24 h or 48 h; figure 2*f*) of Z6 × Z6 pairs indicates that no or few Z6 males copulated with Z6 females after one hour, in marked difference to the results for AL males. More copulations yielding less progeny occurred in Z6 × Z6 pairs during the 24 h period, suggesting that some of these flies showed gametic incompatibility as previously reported [64].

Although AL females were less choosy than Z6 females under white light, they discriminated between AL and Z6 males under red light (figure 2*e*). This indicates that the visual signals had a differential impact on the courtship of AL and Z6 males paired with AL females and that, in the absence of light, AL females were using other cues to choose males. Further studies involving similar Z6 female manipulations are needed to determine which cues are used by AL females to discriminate between male types.

The visual cues perceived during courtship, including the anatomical characteristics of the other fly and its movement, are crucial for mating. The drastic decrease of both courtship frequency and intensity under red light with intact females clearly supports the role of visual cues in sexual interactions; this is further shown by the fact that no such effect was observed with motionless decapitated females (figure 2*a*,*b*; electronic supplementary material, figure S2A). Vision may allow the two partners to precisely synchronize their locomotor activity during courtship [32]. Optomotor-blind *white* mutant males cannot track the moving female and show delayed copulation latency [65–67]. Deprivation of visual cues did not affect the two male types in the same way: under red light, copulation frequency was more affected in Z6 than in AL males (figure 2*e*). This may explain why copulation- and strain-dependent variations of locomotor activity constitute a critical signal in *D. melanogaster* mate recognition and copulation success.

### 4.2. Appendages involved in sexual isolation

#### 4.2.1. Legs and wings

The comparison of behavioural elements that showed differences between copulating and non-copulating Z6 × AL pairs provides a hint about the body parts responsible for the asymmetric sexual isolation. Although few Z6 × AL pairs mated, we were able to take this small sample size into account through our statistical tests. First, Z6 × AL copulating pairs showed an increase in the total duration of female preening. This could conceivably be an acceptance behaviour and indicates the role of female legs in mating (figure 1*b*; [28,68,69]). This is consistent with our other findings: Z6 females without their rear legs (figure 3) showed an increased copulation frequency with AL males. In Z6 females, leg preening was also linked with mating with both kinds of male (electronic supplementary material, figure S3B). Two other behavioural elements, which again could conceivably be associated with female rejection, also varied with mating in Z6 × AL pairs (figure 1*c*). First, Z6 females that mated directed less complete ovipositor extrusion to AL males, as previously reported [28,70]. However, these females kicked AL males more often with their rear legs 3 when compared with non-mating Z6 females. The unexpected stimulatory effect of this behaviour may be due to a strain-specific effect, stimulating AL males and inhibiting Z6 males.

Wings of both sexes are also involved in sexual isolation (figures 3 and 5*c*). When females accept males, they open their wings to permit copulation [71]. This may explain why AL males copulated more often with wing-deprived Z6 females than with intact ones. Wing preening in Z6 females also varied with mating of Z6 × AL pairs and its relative increase seems to stimulate male copulation (electronic supplementary material, figure S3*b*). We do not know why.

Male wings produce acoustic signals that stimulate mating, and all wing parts are not equivalent with regard to copulation success [16,47,50]. Complete bilateral wing ablation in Z6 males drastically reduced their mating frequency with Z6 females (a similar effect was found in AL × AL pairs: figure 3*b* in [30]). Z6 males missing the posterior wing region showed a low mating success, similar to that of intact AL males in a competition experiment. Surprisingly, the bilateral ablation of the distal wing part increased copulation frequency in Z6 males competing with intact AL males for Z6 females. Given that the anterior wing margin is involved in the chemosensory perception of odorant and tastant molecules [48,49], this suggests that wings play a more complex role than hitherto suspected in the exchange of sensory cues during courtship. Males with just one ablated wing need to be compared with intact males to determine whether or not male behaviour is altered because their perception of female cues is involved.

#### 4.2.2. Olfactory and/or auditory cues

Extra-additional sensory stimuli provided by physically separated males affected the copulation frequency of focal Z6 × Z6 pairs: it was decreased with five AL males, but increased with two AL × AL pairs (figure 6*c*). This indicates that some olfactory and auditory signals produced by M flies can stimulate mating in Z flies at a short distance and without contact. Groups of 5 AL males may have sent inhibitory stimuli, including the sex pheromone *cis*-Vaccenyl acetate (cVA; [72]) and/or repulsive vibrations [73], in order to deter male–male courtship, potentially affecting copulation in Z6 × Z6 focal pairs. Conversely, the stimulatory effect induced in Z6 × Z6 focal pairs by the two extra pairs of AL flies could be due to one or several stimulatory pheromone(s) emitted by flies of either sex and perceived at a short distance [74–76]. The mating frequency of Z6 females did not change with or without physical contact with a larger number of Z6 males (figure 6*b*,*c*). Together with the fact that copulation in Z6 × AL focal pairs was not affected by any of these stimuli produced by live flies (figure 6*d*), this reinforces the idea that Z6 females need a very precise sensory picture of their potential mating partner for copulation to occur.

The stimuli perceived in the two-floor mating chambers may mostly act through olfactory perception, and much less by vibration, given that funiculi ablation strongly decreased copulation in both sexes (figure 4*b*; fig. 3*c* in [30]); arista ablation had a smaller or non-existent effect. The effect induced by funiculi ablation was larger in females than in males. The third antennal segment (funiculus) carries many olfactory sensilla, some of which may be involved in the perception of male stimulatory pheromones [75,77]. However, the fact that maxillary palp ablation in females had no effect was surprising, given that these structures carry olfactory receptor neurons responding to cVA [78], a pheromone thought to increase female sexual receptivity [79]. Taken together, these data suggest that some of the sensory cues known to affect the sexual behaviour of M-type flies induce a different effect in Z-type flies, or at least in Z6 × AL pairs.

## 5. Conclusion

While none of our experimental manipulations of appendages, sensory cues and physical contact allowed us to fully rescue the marked sexual isolation seen between Z6 females and AL males, our study adds several pieces to the puzzle of this intensely studied example of incipient speciation. The cosmopolitan distribution of current M strains, as opposed to the geographically restricted area of Z strains, suggests that the survival of contemporary Z strains, considered to be derived from the ancestral form of the *D. melanogaster* species [80], depends on its tight adaptation to a very specific environment. Our next challenge will be to explore whether or not the survival of Z strains among a world of M strains is related to the high choosiness of Z females, thereby preventing the introgression of M paternal genes into the population.

## Supplementary Material

Suppl. Fig. 1: Two-floors mating chamber

## Supplementary Material

Suppl. Fig. 2: Locomotor activity indices measured with single flies

## Supplementary Material

Suppl. Fig. 3: Body parts involved in female preening behaviour

## Supplementary Material

Suppl. Fig. 4: Effect of light condition.

## Supplementary Material

Figure_1_A

## Supplementary Material

Figure_1_B_C_&_Suppl_Figure_S3

## Supplementary Material

Figure_2_&_Suppl_Figure_S4.xlsx

## Supplementary Material

Figure_3

## Supplementary Material

Figure_4

## Supplementary Material

Figure_5

## Supplementary Material

Figure_6

## Supplementary Material

Suppl_Figure_2

## Data accessibility

All experimental data can be found in the electronic supplementary material (XLSX files) and at the Dryad Digital Repository (http://dx.doi.org/10.5061/dryad.sg095) [81].
