# Supplementary figures and images for: Behavioural elements and sensory cues involved in sexual isolation between Drosophila melanogaster strains
Source: R Soc Open Sci. 2018 May 9;5(5):172060. doi: 10.1098/rsos.172060 (PMC5990781; doi:10.1098/rsos.172060)

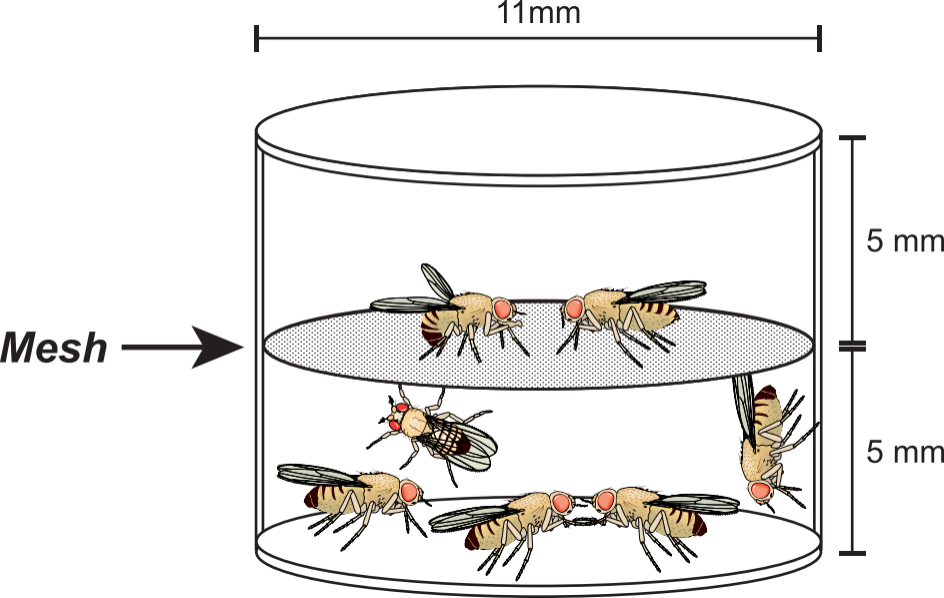

Supplement: Suppl. Fig. 1: Two-floors mating chamber [file rsos172060supp1.pdf]

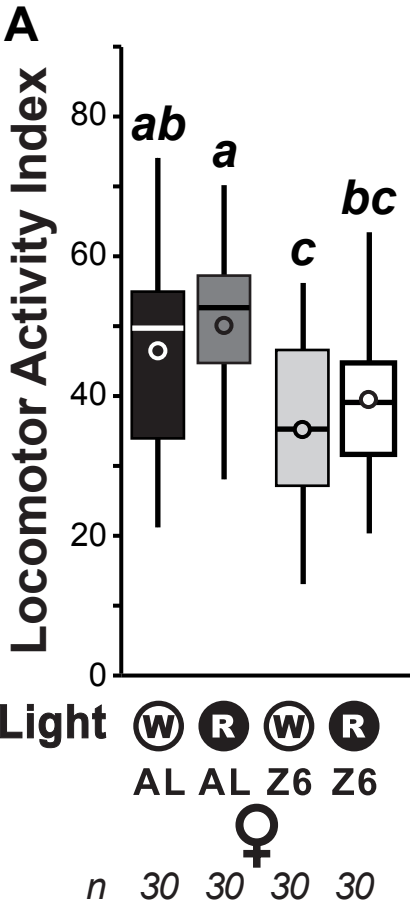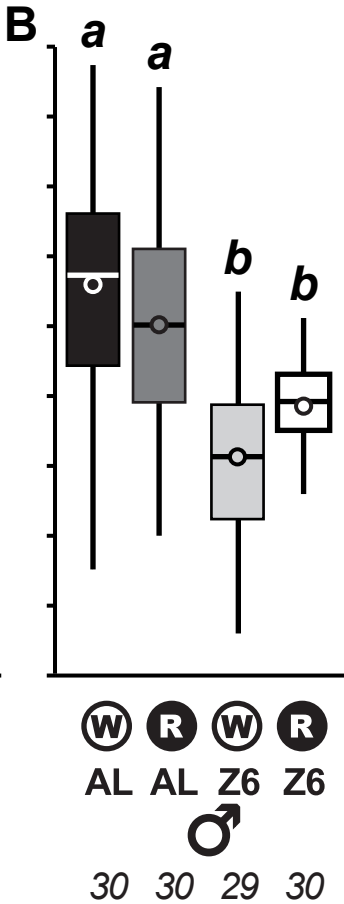

Supplement: Suppl. Fig. 2: Locomotor activity indices measured with single flies [file rsos172060supp2.pdf]

A

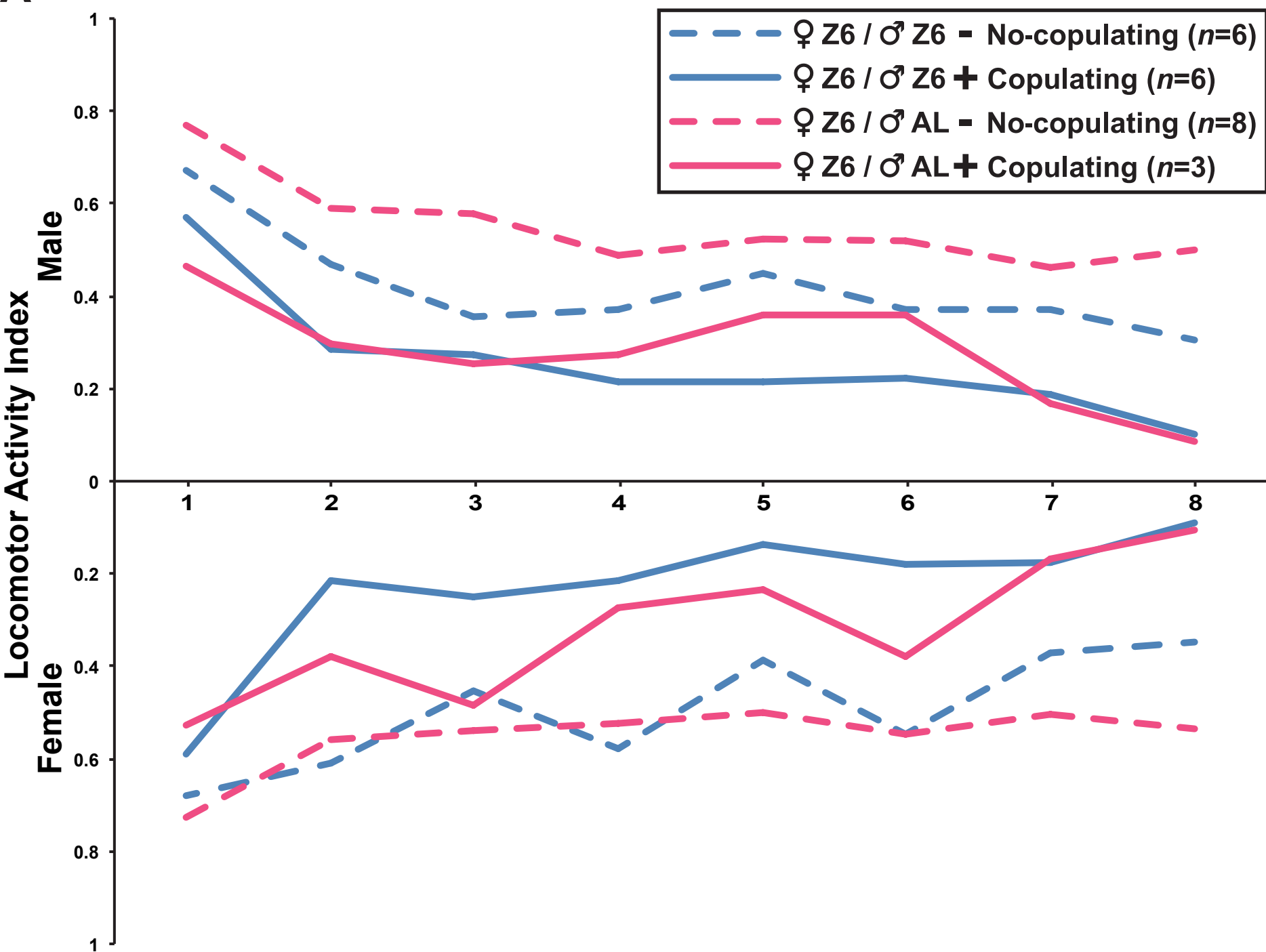

B

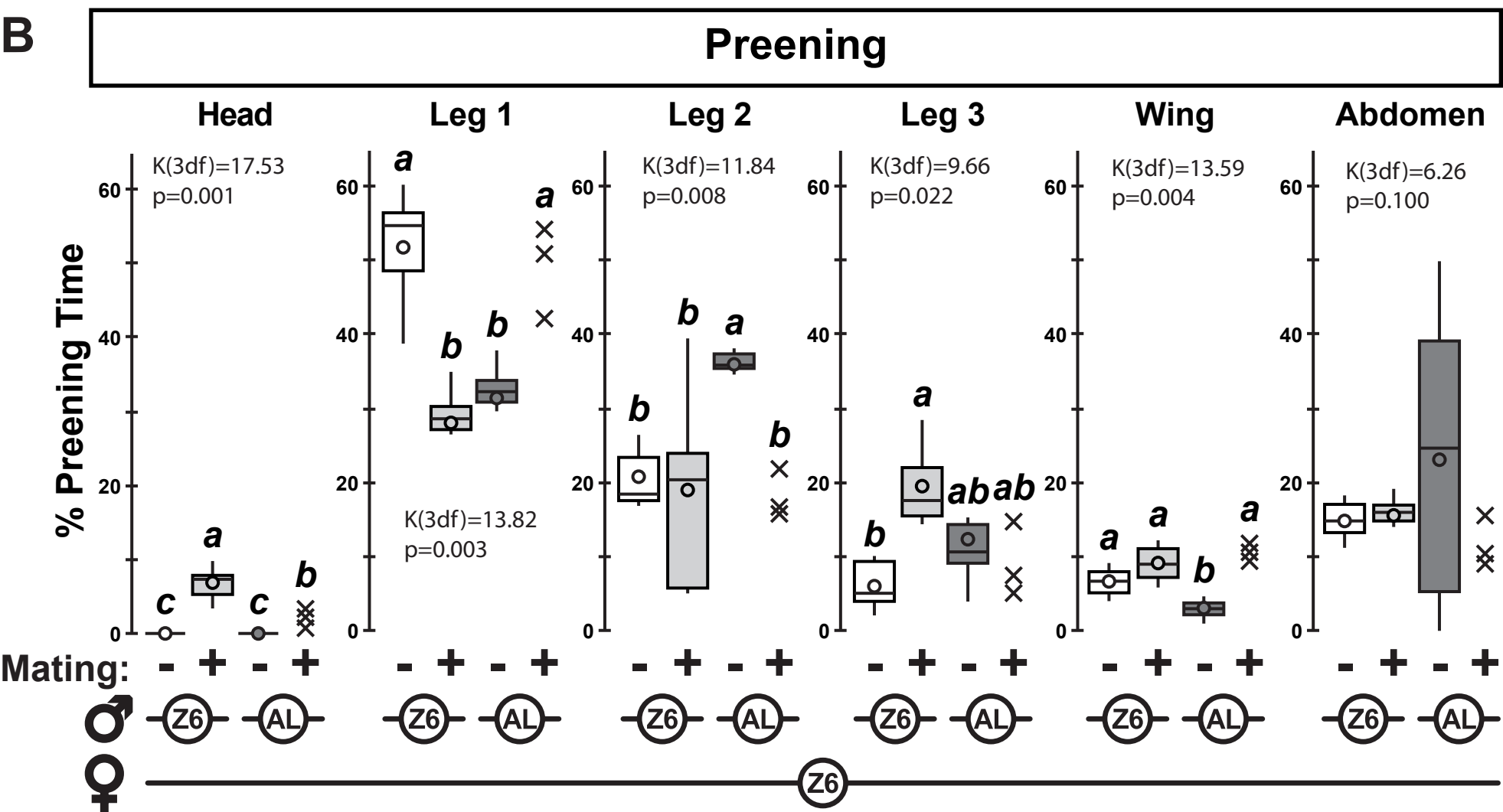

Supplement: Suppl. Fig. 3: Body parts involved in female preening behaviour [file rsos172060supp3.pdf]
