# Supplementary material for: Behavioural elements and sensory cues involved in sexual isolation between Drosophila melanogaster strains
Source: R Soc Open Sci. 2018 May 9;5(5):172060. doi: 10.1098/rsos.172060 (PMC5990781; doi:10.1098/rsos.172060)
Supplement: Suppl. Fig. 4: Effect of light condition. [file rsos172060supp4.pdf]

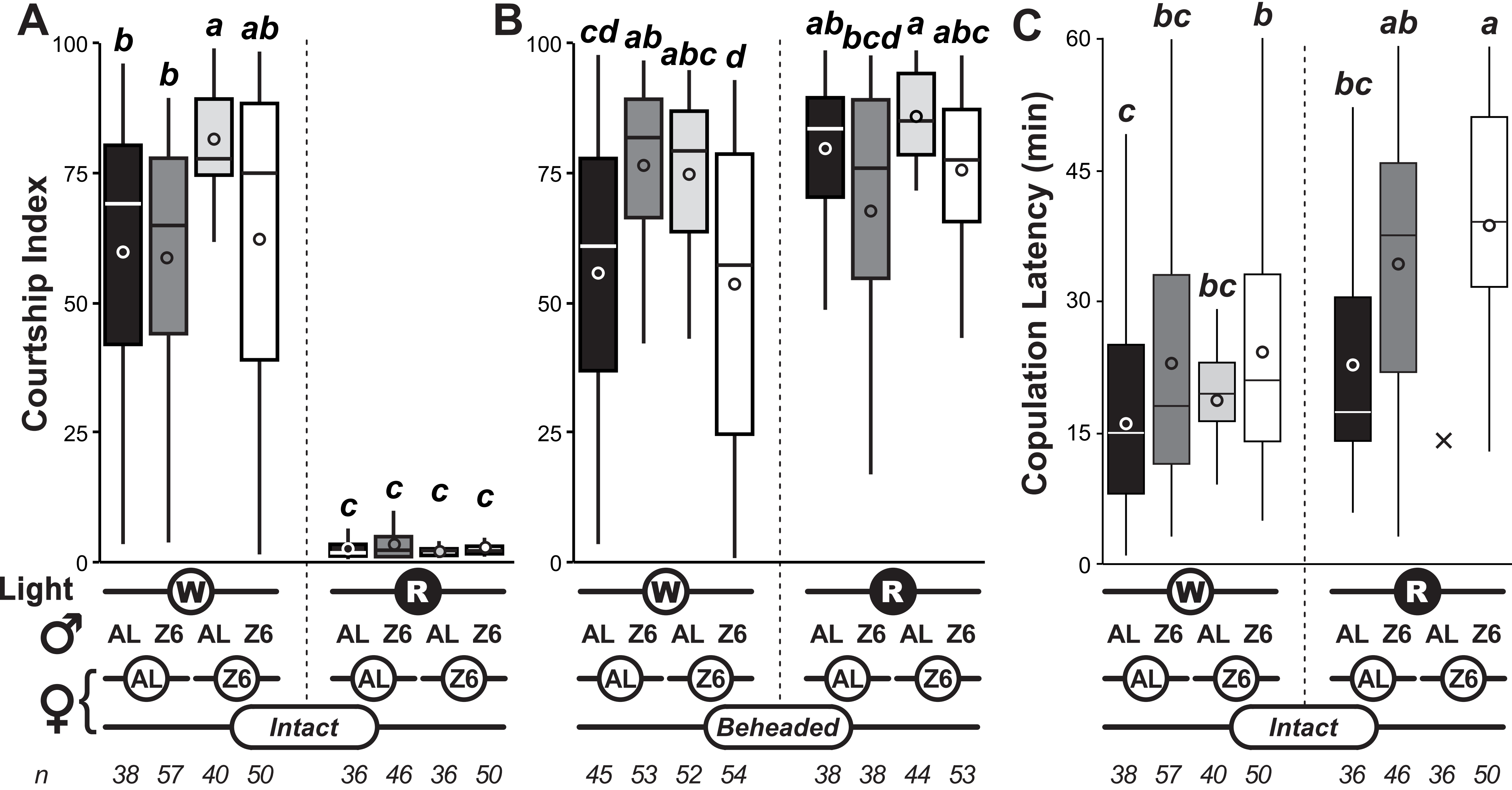

A
B
C
Courtship Index
Copulation Latency (min)
b
b
a
ab
c
c
c
c
cd
ab
abc
d
ab
bcd
a
abc
bc
b
bc
ab
bc
a
Light
W
R
♂
AL Z6 AL Z6
♀
AL
Z6
Intact
Beheaded
n
38 57 40 50 36 46 36 50
45 53 52 54 38 38 44 53
38 57 40 50 36 46 36 50
